# Supplementary figures and images for: A mutant methionyl-tRNA synthetase-based toolkit to assess induced-mesenchymal stromal cell secretome in mixed-culture disease models
Source: Stem Cell Res Ther. 2023 Oct 5;14:289. doi: 10.1186/s13287-023-03515-0 (PMC10557244; doi:10.1186/s13287-023-03515-0)

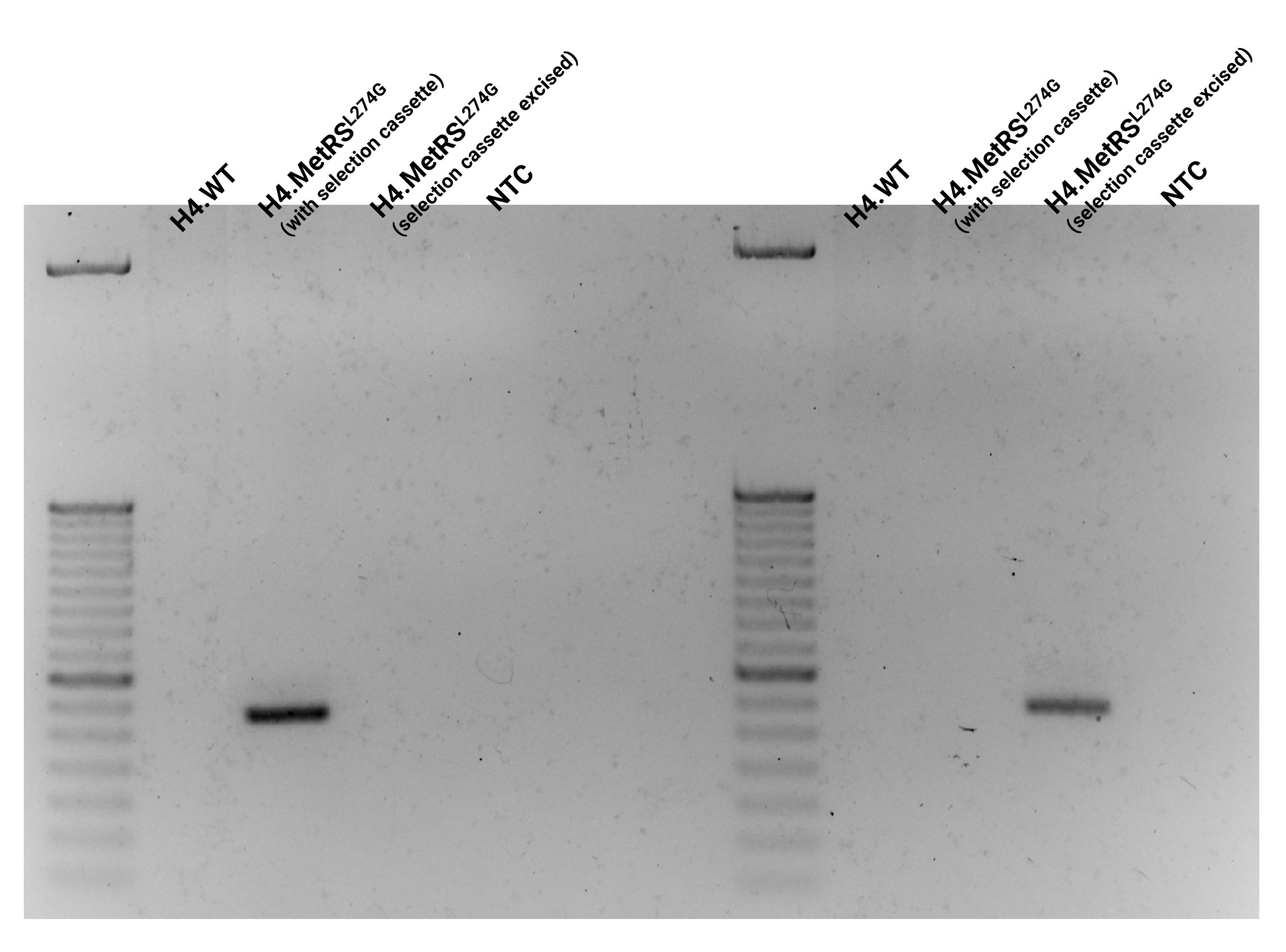

Supplement: Supplementary file 1 — Additional file 1: Fig. S1. Uncropped agarose gel from Figure 1B. [file 13287_2023_3515_MOESM1_ESM.png]

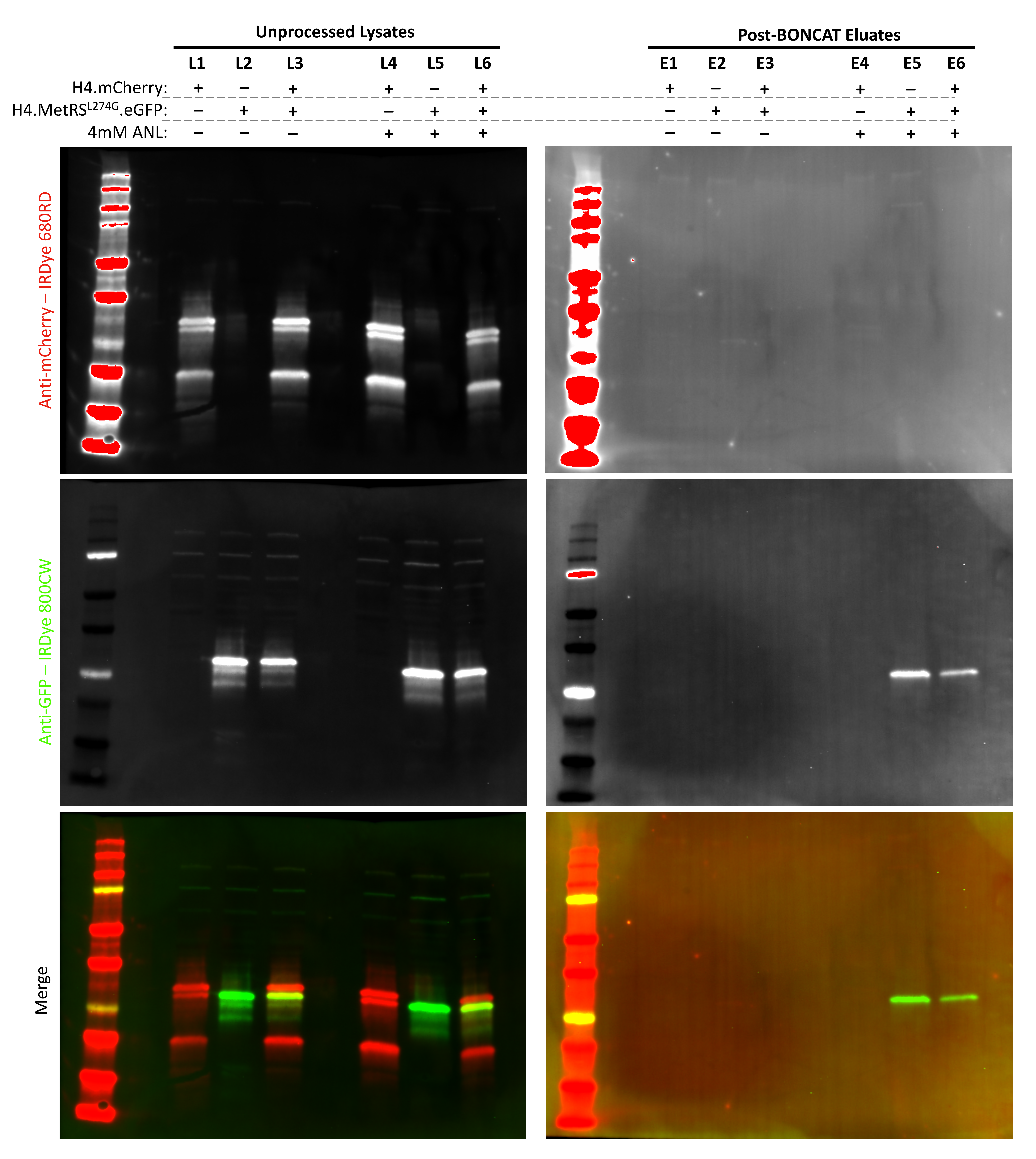

Supplement: Supplementary file 2 — Additional file 2: Fig. S2. Uncropped WB membranes from Figure 2C. [file 13287_2023_3515_MOESM2_ESM.png]

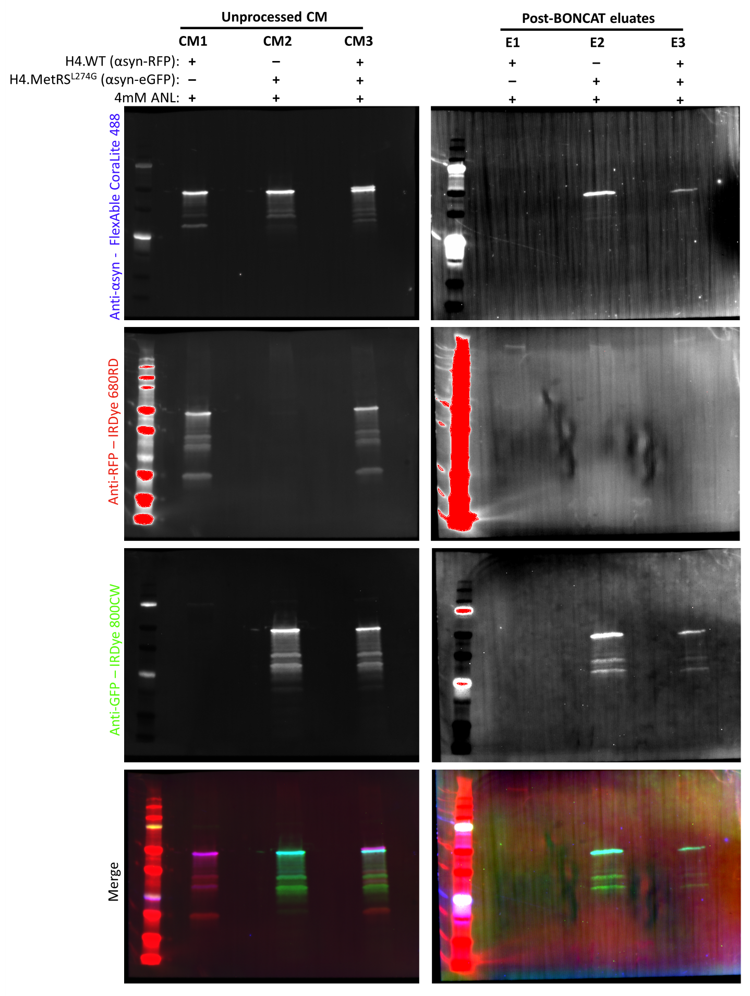

Supplement: Supplementary file 3 — Additional file 3: Fig. S3. Uncropped WB membranes from Figure 3C. [file 13287_2023_3515_MOESM3_ESM.png]

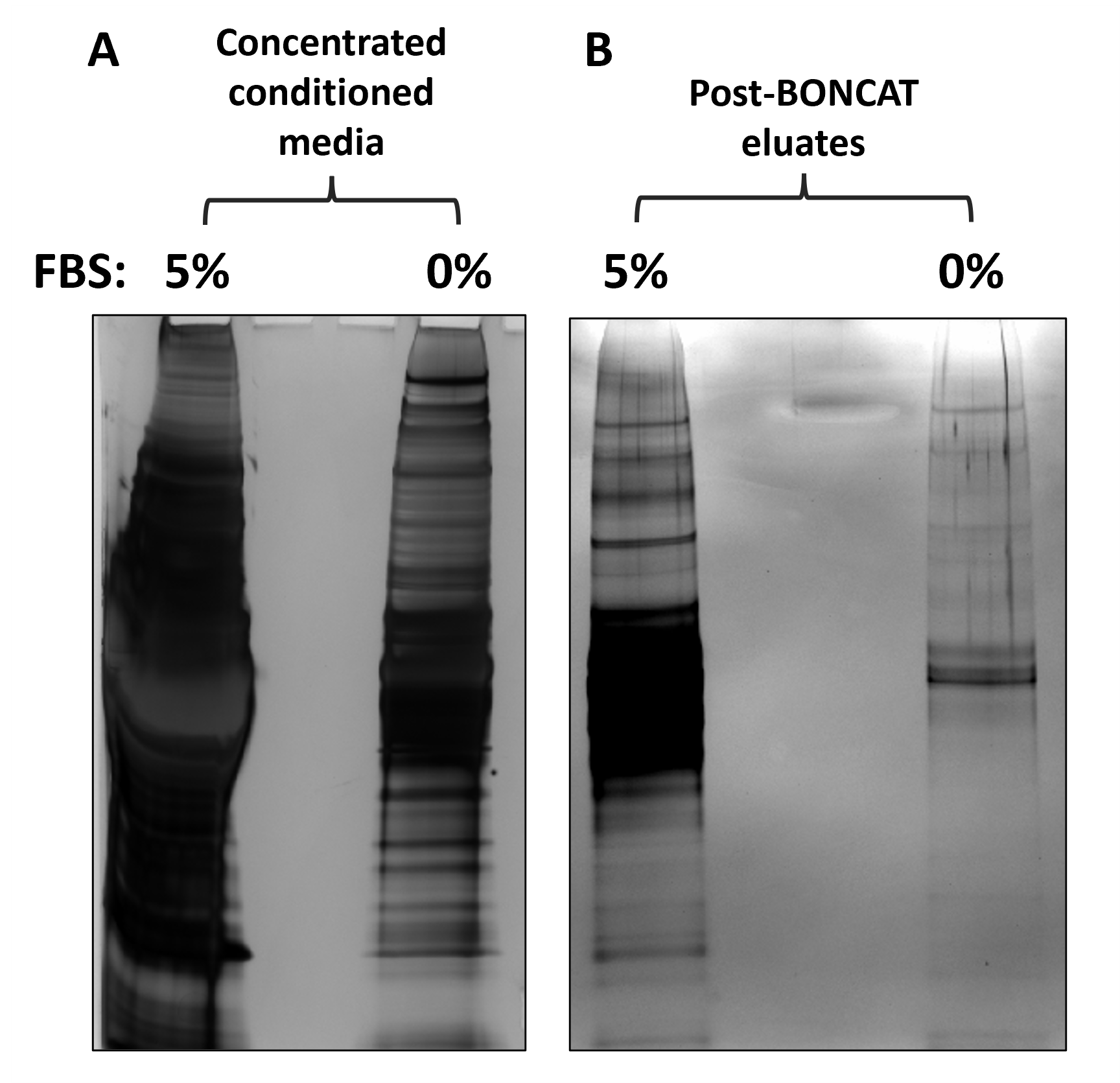

Supplement: Supplementary file 4 — Additional file 4: Fig. S4. H4.MetRSL274G cells were cultured in 4mM ANL-supplemented media either serum-free or with 5% FBS. A) Silver stain for concentrated media shows large albumin band masking the majority of proteins in 5% FBS-containing media. B) Following BONCAT processing and affinity purification, albumin band is greatly diminished, allowing detection of many lower abundance proteins. [file 13287_2023_3515_MOESM4_ESM.png]

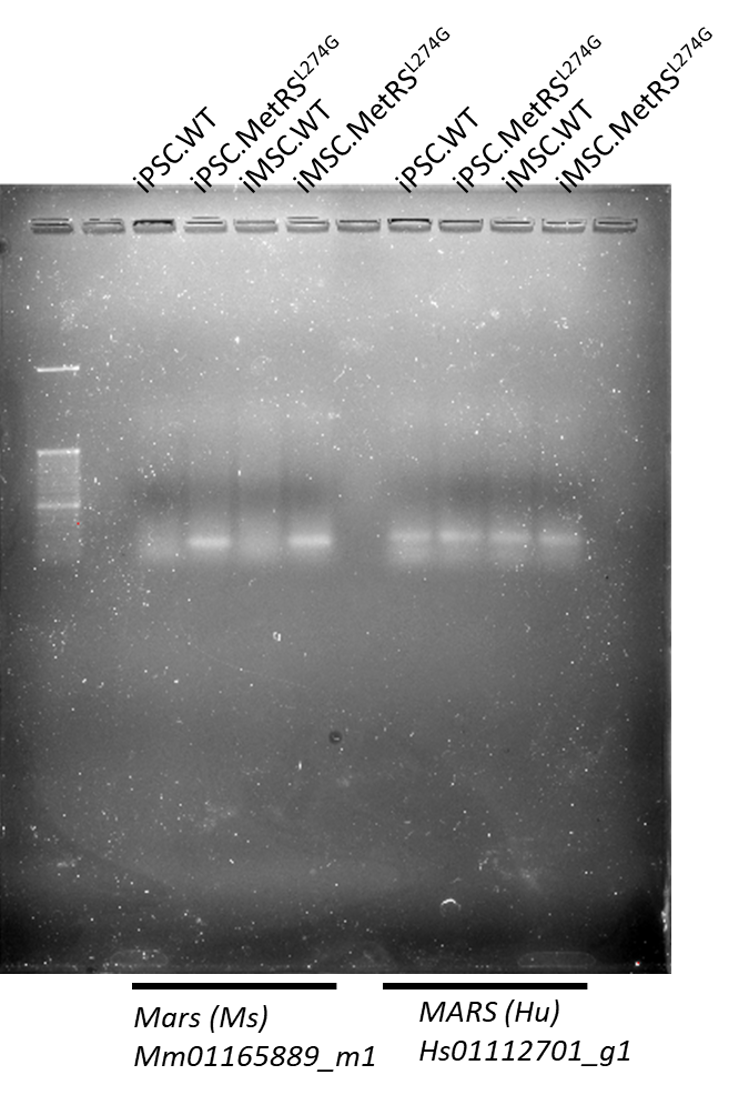

Supplement: Supplementary file 5 — Additional file 5: Fig. S5. Uncropped agarose gel from Figure 5C. [file 13287_2023_3515_MOESM5_ESM.png]
